# Citral modulates virulence factors in methicillin-resistant *Staphylococcus aureus*

**DOI:** 10.1038/s41598-021-95971-y

**Published:** 2021-08-13

**Authors:** Hellen Braga Martins Oliveira, Nathan das Neves Selis, Beatriz Almeida Sampaio, Manoel Neres Santos Júnior, Suzi Pacheco de Carvalho, Jéssica Bomfim de Almeida, Palloma Porto Almeida, Icaro Bonyek Santos da Silva, Caline Novais Teixeira Oliveira, Thamara Louisy Santos Brito, Letícia de Oliveira da Silva, Mariana Morais Teixeira, Hanna Izadora Laís Novaes Coelho, Camila Dutra Barbosa, Yasmin Monara Ferreira de Sousa Andrade, Rafaela de Souza Bittencourt, Jully Chayra Santos Viana, Guilherme Barreto Campos, Jorge Timenetsky, Ana Paula T. Uetanabaro, Regiane Yatsuda, Lucas Miranda Marques

**Affiliations:** 1 Universidade Estadual de Santa Cruz, Rod. Jorge Amado, Km 16, Salobrinho, Ilhéus, Bahia 45662-900 Brazil; 2 Instituto Multidisciplinar em Saúde, Universidade Federal da Bahia, Rua Hormindo Barros, 58, Candeias, Vitória da Conquista, Bahia 45029-094 Brazil; 3 Departamento de Biologia Geral, Universidade Federal de Viçosa, Av. Peter Henry Rolfs S/N. Campus Universitário, Viçosa, Minas Gerais CEP 36570-000 Brazil; 4 Instituto Gonçalo Muniz, Fundação Oswaldo Cruz, Rua Waldemar Falcão, 121, Candeal, Salvador, Bahia 40296-710 Brazil; 5 Instituto de Ciências Biomédicas, Universidade de São Paulo, Avenida Professor Lineu Prestes, 2415, Butantã, São Paulo 05508-900 Brazil

**Keywords:** Microbiology, Pathogens

## Abstract

Methicillin-resistant *Staphylococcus aureus* (MRSA) is responsible for high morbidity and mortality rates. Citral has been studied in the pharmaceutical industry and has shown antimicrobial activity. This study aimed to analyze the antimicrobial activity of citral in inhibiting biofilm formation and modulating virulence genes, with the ultimate goal of finding a strategy for treating infections caused by MRSA strains. Citral showed antimicrobial activity against MRSA isolates with minimum inhibitory concentration (MIC) values between 5 mg/mL (0.5%) and 40 mg/mL (4%), and minimum bactericidal concentration (MBC) values between 10 mg/mL (1%) and 40 mg/mL (4%). The sub-inhibitory dose was 2.5 mg/mL (0.25%). Citral, in an antibiogram, modulated synergistically, antagonistically, or indifferent to the different antibiotics tested. Prior to evaluating the antibiofilm effects of citral, we classified the bacteria according to their biofilm production capacity. Citral showed greater efficacy in the initial stage, and there was a significant reduction in biofilm formation compared to the mature biofilm. qPCR was used to assess the modulation of virulence factor genes, and *ica*A underexpression was observed in isolates 20 and 48. For *ica*D, *seg*, and *sei*, an increase was observed in the expression of ATCC 33,591. No significant differences were found for *eta* and *etb*. Citral could be used as a supplement to conventional antibiotics for MRSA infections.

## Introduction

*Staphylococcus aureus* is an important and potentially lethal opportunistic pathogen. These bacteria have high virulence and the ability to acquire resistance mechanisms and pathogenic characteristics. *S. aureus* is normally associated with various infections acquired in the community and in hospitals^1–3^.

Widespread and indiscriminate use of antibiotics can lead to the selection and antimicrobial resistance of bacterial isolates. The detection of antibiotic-resistant pathogens is relevant for therapeutic purposes, as well as to prevent the spread of resistant strains^4^. The *S. aureus* strain resistant to almost all β-lactam antibiotics, determined by a chromosomal gene *mecA* that encodes altered PBPs (PPB2a or PBP2'), is referred to as methicillin-resistant *Staphylococcus aureus* (MRSA) and can be found in hospital settings (HA-MRSA), as well as in the community (CA-MRSA)^5^. Infections caused by these bacteria are linked to higher mortality rates and higher treatment costs for an overburdened health system compared to infections caused by strains of *S. aureus* sensitive to methicillin (MSSA)^6–8^.

The virulence potential of different isolates of *S. aureus* is determined by the presence or absence of virulence genes that encode staphylococcal enterotoxins (*ses*), Panton-Valentine leukocidin (*pvl*), exfoliatins (*eta* and *etb*), hemolysins, and other exotoxins. These genes provide bacteria with the ability to infect and colonize^9,10^, resulting in food poisoning and other types of infections in humans and animals.

Because of bacterial resistance, a better understanding of the factors involved in the pathogenicity of *S. aureus* is important for developing alternative treatments. Thus, the choice of antibiotics for treating infections caused by this microorganism is limited^11^. In the search for an alternative to antibiotics, natural products have been studied, including essential oils. Essential oils are volatile secondary metabolites composed of complex mixtures of organic substances, characterized by a strong odor, and formed in response to stressors. These substances feature actions such as passage through a cell wall and plasma membrane, which can affect the cytotoxic properties of bacterial structures, in addition to presenting therapeutic protective properties against oxidation and deterioration processes caused by microorganisms^12–14^.

Among the major components extracted from the removal of essential oils from edible aromatic plants, citral (3,7-dimethyl-2,6-octadienal) is a natural mixture of geranial (trans-citral) and neral (cis-citral), which are two acyclic monoterpenic aldehydes and isomers, found in a variety of plants, such as in melissa (*Melissa officinalis*), lemongrass (*Cymbopogon citratus*), and verbena (*Verbena officinalis*)^15,16^.

Various studies have shown the antimicrobial activity of components of essential oils, such as citral, on strains of *S. aureus*, inhibiting biofilm formation or altering virulence factors. It is known that essential oils and major components can act to deregulate virulence genes, in addition to antibacterial, antifungal, analgesic, antispasmodic, and antiparasitic activities, in addition to fighting nerve disorders^15,17,18^. Despite the antibacterial properties of this oil against gram-positive and gram-negative bacteria, its prolonged use has not shown resistance^19,20^.

In a previous study, we analyzed the anti-inflammatory action of citral using an air pouch model in 48 male BALB/c mice infected and/or treated with citral. A positive effect was observed in reducing the microorganism, in addition to a significant decrease in the levels of TNF-α in mice treated with citral^21^. Thus, this study considers a new therapeutic alternative for infections triggered by MRSA, analysing the effect of citral on the antimicrobial effect of antibiotics, inhibition of biofilm formation, and modulation of the expression of virulence factors. This study is not only relevant but pertinent, as there are few studies involving citral and MRSA.

## Results

The minimum inhibitory concentration (MIC) and minimum bactericidal concentration (MBC) data are listed in Table 1. The lowest concentration that inhibited bacterial growth was 5 mg/mL (0.5%), whereas the highest concentration was 40 mg/mL (4%). The bactericidal concentrations varied between 10 mg/mL (1%) and 40 mg/mL (4%). Of the nine MRSA strains tested, five showed the same concentration in both tests. Using the growth curve, it was possible to determine the concentration that would decrease bacterial growth but would not kill the isolates. After defining MIC and MBC, the subinhibitory concentration of citral was determined to verify the modulation of virulence factors without affecting the viability of microorganisms. Therefore, tests were performed with the concentrations 40 mg/mL (4%), 20 mg/mL (2%), 10 mg/mL (1%), 5 mg/mL (0.5%), 2.5 mg/mL (0.25%), 1.2 mg/mL (0.12%), and 0.6 mg/mL (0.06%), with a concentration of 2.5 mg/mL being determined for achieving the curve (Table 1).

**Table 1:** MIC, MBC, and subinhibitory dose values for isolates of MRSA treated with citral.

Isolates	MIC (mg/mL)	MBC (mg/mL)	Subinhibitory dose (mg/mL)
18	10	20	2.5
20	10	40	2.5
27	10	10	2.5
33	20	20	2.5
48	10	10	2.5
52	40	40	2.5
80	5	40	2.5
137	5	20	2.5
ATCC 33591	10	10	2.5

*MIC* minimum inhibitory concentrations, *MBC* minimum bactericidal concentrations.

The analysis of modulating activity was compared with the Clinical & Laboratory Standards Institute (CLSI) manual (2020), with the following resistance halos: ≤ 28 for ampicillin, ≤ 15 for ciprofloxacin, ≤ 14 for clindamycin, ≤ 13 for erythromycin, ≤ 21 for oxacillin, and ≤ 14 for tetracycline. The inhibition zone (mm) and fractional inhibitory concentration were measured after 24 h of incubation. Our results showed that 5 mg/mL (0.5%) citral was able to modulate the antimicrobial activity of different antibiotics through synergistic, antagonistic, or indifferent actions. In the inhibition zone (mm), the results were statistically significant for strain 80 with ciprofloxacin, for strains 52 and 80 with the antibiotic erythromycin, strains 80 and ATCC 33591 with oxacillin, and for strain 52 with the antibiotic tetracycline (*P* < 0.05). Citral acted antagonistically with the antibiotic ampicillin for strains 52 and 80, with the use of ciprofloxacin for strain 52, oxacillin in isolate 20, and tetracycline in isolate 18. All tested strains remained resistant to the antibiotics tested (Table 2). In the analysis of the fractional inhibitory concentration (μg/mL), the results were statistically significant for strain 80 for oxacillin (Table 2). Comparing the modulating effect of citral on antibiotic activity when measured via the disk diffusion and broth microdilution methods, different results were obtained. We observed that only strain 80 maintained its results for the antibiotic oxacillin. Based on our experiment, the checkerboard method is more sensitive for detecting synergy and disk-diffusion may serve as an initial screening method for the detection of potential synergies.

**Table 2:** Average of triplicate with three independent repetitions of inhibition zone (mm) values and MIC (μg/mL) values for antibacterial modulating activity of citral 5 mg/mL (0.5%) on antibiotics ampicillin (Amp), ciprofloxacin (Cipro), clindamycin (Clinda), erythromycin (Ery), oxacillin (Oxa), tetracycline (Tet) on MRSA (18, 20, 27, 33, 48, 52, 80, 137) and ATCC 33591 strains.

MRSA	Treatment (μg/mL)																	
	Amp	Amp + citral	p-value^a^	Cipro	Cipro + citral	p-value^a^	Clinda	Clinda + citral	p-value^a^	Erytro	Erytro + citral	p-value^a^	Oxa	Oxa + citral	p-value^a^	Tetra	Tetra + citral	p-value^a^
18	0.5 ± 0.0	0.5 ± 0.0	> 0.9999	0.7 ± 0.0	0.7 ± 0.0	> 0.9999	0.8 ± 0.3	0.7 ± 0.3	0.5185	2 ± 0.3	2 ± 0.3	> 0.9999	2 ± 0.0	2 ± 0.0	> 0.9999	0.5 ± 0.0	1 ± 0.0	> 0.9999
20	0.5 ± 0.0	0.5 ± 0.0	> 0.9999	1 ± 0.0	1 ± 0.0	> 0.9999	1 ± 0.0	1 ± 0.0	> 0.9999	2 ± 0.0	2 ± 0.0	> 0.9999	8 ± 0.0	8 ± 0.0	> 0.9999	1 ± 0.0	1 ± 0.0	> 0.9999
27	0.5 ± 0.0	0.5 ± 0.0	> 0.9999	1 ± 0.0	1 ± 0.0	> 0.9999	1 ± 0.0	1.3 ± 0.0	0.3739	2 ± 0.0	2 ± 0.6	> 0.9999	8 ± 0.0	8 ± 0.0	> 0.9999	1 ± 0.0	1 ± 0.0	> 0.9999
33	0.5 ± 0.0	0.5 ± 0.0	> 0.9999	2 ± 0.0	2 ± 0.0	> 0.9999	2 ± 0.0	2 ± 0.0	> 0.9999	2 ± 0.0	2 ± 0.0	> 0.9999	2 ± 0.0	2 ± 0.0	> 0.9999	8 ± 0.0	8 ± 0.0	> 0.9999
48	1 ± 0.0	0.8 ± 0.3	0.3739	1 ± 0.0	0.8 ± 0.3	0.3739	1 ± 0.0	1 ± 0.3	> 0.9999	4 ± 0.0	4 ± 0.0	> 0.9999	5.3 ± 0.0	4 ± 0.0	> 0.9999	1 ± 0.0	1 ± 0.0	> 0.9999
52	0.12 ± 0.0	0.5 ± 0.0	> 0.9999	0.5 ± 0.0	2 ± 0.0	> 0.9999	0.5 ± 0.0	0.7 ± 0.0	0.3739	5.3 ± 0.0	4 ± 0.3	0.3739	2 ± 2.3	2 ± 0.0	> 0.9999	2 ± 0.0	1 ± 0.0	> 0.9999
80	0.5 ± 0.0	0.5 ± 0.0	> 0.9999	64 ± 0.0	2 ± 0.0	> 0.9999	1.7 ± 0.0	1 ± 0.0	0.1161	4 ± 0.6	2 ± 0.0	> 0.9999	64 ± 0.0	6 ± 0.0	** < 0.0001**	1.3 ± 0.6	0.8 ± 0.4	0.2508
137	0.25 ± 0.0	0.12 ± 0.0	> 0.9999	2 ± 0.0	2 ± 0.0	> 0.9999	2 ± 0.0	2 ± 0.0	> 0.9999	126 ± 0.0	126 ± 0.0	> 0.9999	0 ± 0.0	8 ± 0.0	0.1161	1.7 ± 0.6	1.3 ± 0.6	0.5185
ATCC 33591	0.7 ± 0.3	1 ± 0.0	0.1161	0.5 ± 0.3	0.5 ± 0.0	> 0.9999	0.5 ± 0.0	0.5 ± 0.0	> 0.9999	126 ± 0.0	126 ± 0.0	> 0.9999	64 ± 0.0	2 ± 0.0	> 0.9999	8 ± 0.0	2 ± 0.0	> 0.9999

MRSA	Treatment (mm)																	
	Amp	Amp + citral	p-value^a^	Cipro	Cipro + citral	p-value^a^	Clinda	Clinda + citral	p-value^a^	Erytro	Erytro + citral	p-value^a^	Oxa	Oxa + citral	p-value^a^	Tetra	Tetra + citral	p-value^a^
18	1.9 ± 0.1	2.1 ± 0.1	0.1011	3.9 ± 0.1	3.9 ± 0.1	0.6778	3.9 ± 0.1	3.9 ± 0.1	0.6779	3.6 ± 0.1	3.7 ± 0.2	0.3295	3 ± 0.0	3 ± 0.0	> 0.9999	4 ± 0.0	3.4 ± 0.1	**0.0010**
20	2.2 ± 0.1	2.5 ± 0.1	0.0390	3.5 ± 0.2	3.7 ± 0.1	0.2378	3.1 ± 0.1	3.2 ± 0.2	0.1890	3.1 ± 0.1	3.1 ± 0.1	0.5185	2 ± 0.1	1.7 ± 0.0	**0.0013**	3.5 ± 0.3	3.7 ± 0.2	0.3045
27	1.9 ± 0.2	2.8 ± 0.2	0.1144	3.7 ± 0.3	3.7 ± 0.1	0.8416	3.3 ± 0.3	3.1 ± 0.3	0.5659	3 ± 0.1	3 ± 0.1	> 0.9999	1.7 ± 0.1	1.9 ± 0.1	0.0668	3.5 ± 0.2	3.7 ± 0.2	0.2508
33	2.4 ± 0.1	2.6 ± 0.1	0.1481	2.4 ± 0.2	2.6 ± 0.1	0.1890	2 ± 0.2	2.1 ± 0.1	0.1890	3.1 ± 0.0	3 ± 0.1	0.1161	3.1 ± 0.2	3 ± 0.1	0.3487	0.9 ± 0.1	1 ± 0.1	0.1011
48	1.5 ± 0.1	1.8 ± 0.2	0.1925	3.2 ± 0.2	3.7 ± 0.3	0.0290	3.4 ± 0.2	3.5 ± 0.3	0.0289	2.7 ± 0.1	3 ± 0.0	0.1583	2.1 ± 0.2	1.9 ± 0.1	0.0647	3.4 ± 0.1	3.5 ± 0.0	0.1583
52	3.3 ± 0.1	2.6 ± 0.1	**0.0005**	4.8 ± 0.2	2 ± 0.0	** < 0.0001**	4.5 ± 0.1	4 ± 0.2	0.0179	1.6 ± 0.1	2.2 ± 0.2	**0.0062**	2.8 ± 0.2	2.2 ± 0.1	0.0120	2.8 ± 0.1	3.2 ± 0.1	**0.0010**
80	2.6 ± 0.1	2 ± 0.0	** < 0.0001**	0 ± 0.0	2.4 ± 0.2	** < 0.0001**	2.9 ± 0.1	3.1 ± 0.0	0.0075	2.9 ± 0.1	3.1 ± 0.0	**0.0075**	0 ± 0.0	2.2 ± 0.2	** < 0.0001**	3.9 ± 0.1	3.9 ± 0.2	> 0.9999
137	2.8 ± 0.1	3 ± 0.1	0.0075	2.6 ± 0.1	2.7 ± 0.1	0.2508	2.6 ± 0.1	2.7 ± 0.1	0.2508	0 ± 0.0	0 ± 0.0	> 0.9999	1.3 ± 0.2	1.5 ± 0.1	0.1011	2.8 ± 0.2	3 ± 0.2	0.3982
ATCC 33591	1.1 ± 0.1	1.4 ± 0.1	0.0158	4.1 ± 0.2	4.5 ± 0.0	0.0514	4.1 ± 0.2	4.5 ± 0.0	0.0514	0 ± 0.0	0 ± 0.0	> 0.9999	0 ± 0.0	2.5 ± 0.1	** < 0.0001**	1.3 ± 0.0	2 ± 0.0	> 0.9999

^a^Mann–Whitney one-tailed test was used.

Bold: data with statistical significance.

**Figure 1:**
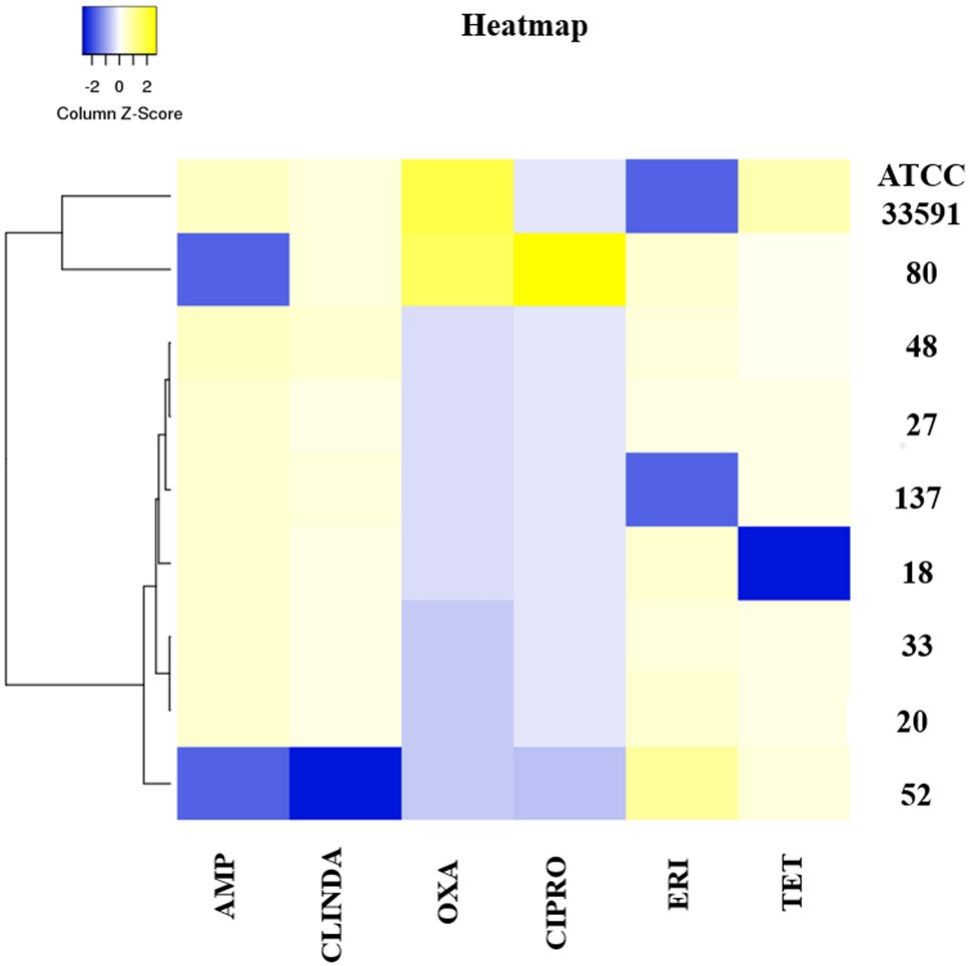
Heatmapper platform formed two clusters according of differential evaluation of the modulating activity at citral 5 mg/mL (0.5%) on antibiotics ampicillin (AMP), ciprofloxacin (CIPRO), clindamycin (CLINDA), erythromycin (ERY), oxacillin (OXA), tetracycline (TET) on MRSA (18, 20, 27, 33, 48, 52, 80, 137) and ATCC 33591 strains. The yellow segments represent greater modulation of 0.5% citral on antibiotics, and the blue segments represent less modulation.

Antibiograms were also analyzed using the heatmapper platform (http://www.heatmapper.ca/expression/), and differentiated modulating activity patterns induced by different strains of MRSA were observed. The heatmap separated the isolates into three distinct clusters (cluster 1: ATCC 33591 and strain 80; cluster 2: strains 48, 27, 137, 18, 33, and 20; cluster 3: strain 52) and, together with the qPCR data, showed different expression patterns induced by different strains of MRSA (Fig. 1).

**Figure 2:**
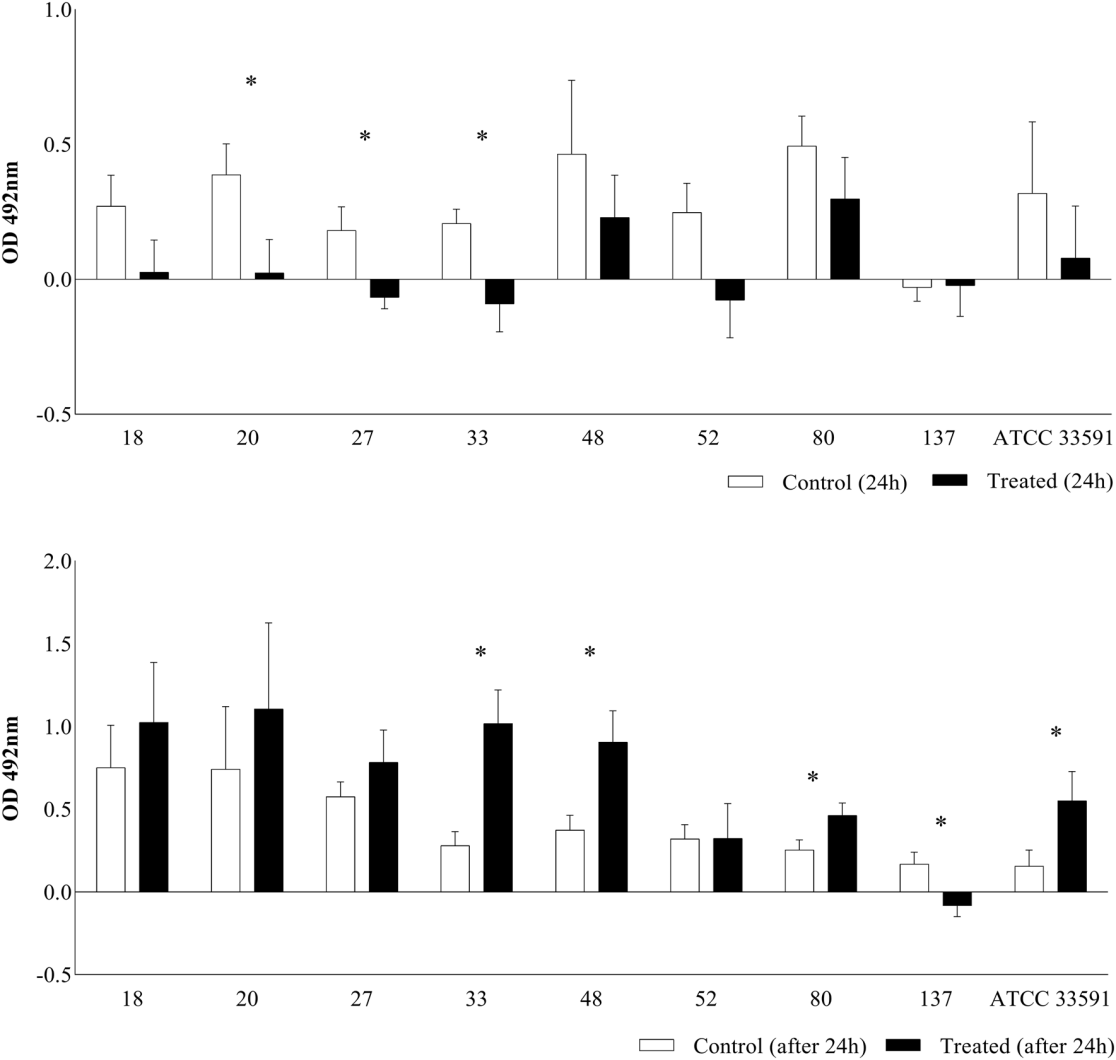
Biofilm formation capacity of the MRSA strains (18, 20, 27, 33, 48, 52, 80, 137) and ATCC 33591 control treated with 25 mg/mL (2.5%) citral for 24 h and with the addition of 2.5% citral after 24 h of the formed biofilm represented in a quantitative way. The experiment was carried out in quadruplicate with two independent experiments. Significant difference at *P* ≤ 0.05 (*).

Biofilm formation capacity of the MRSA were represented in a quantitative way in MRSA isolates using citral in the initial phase of biofilm formation from 0 to 24 h, along with the addition of citral in the mature phase of the biofilm, corresponding to 24 h after inoculation. The concentration of 25 mg/mL (2.5%) of citral was more effective in isolates with citral added in the initial phase of biofilm formation than in the mature phase. In the initial phase, there was a significant reduction in biofilm formation, as isolate 20 (93.6% reduction), and thus isolates 27 (62.5% reduction) and 33 (55.71% reduction). In the mature phase, isolate 137 (51.2% reduction) after 24 h of growth was the only isolate showing a positive result. Thus, it was observed that using citral in the initial phase of biofilm formation presented better results (Fig. 2).

**Figure 3:**
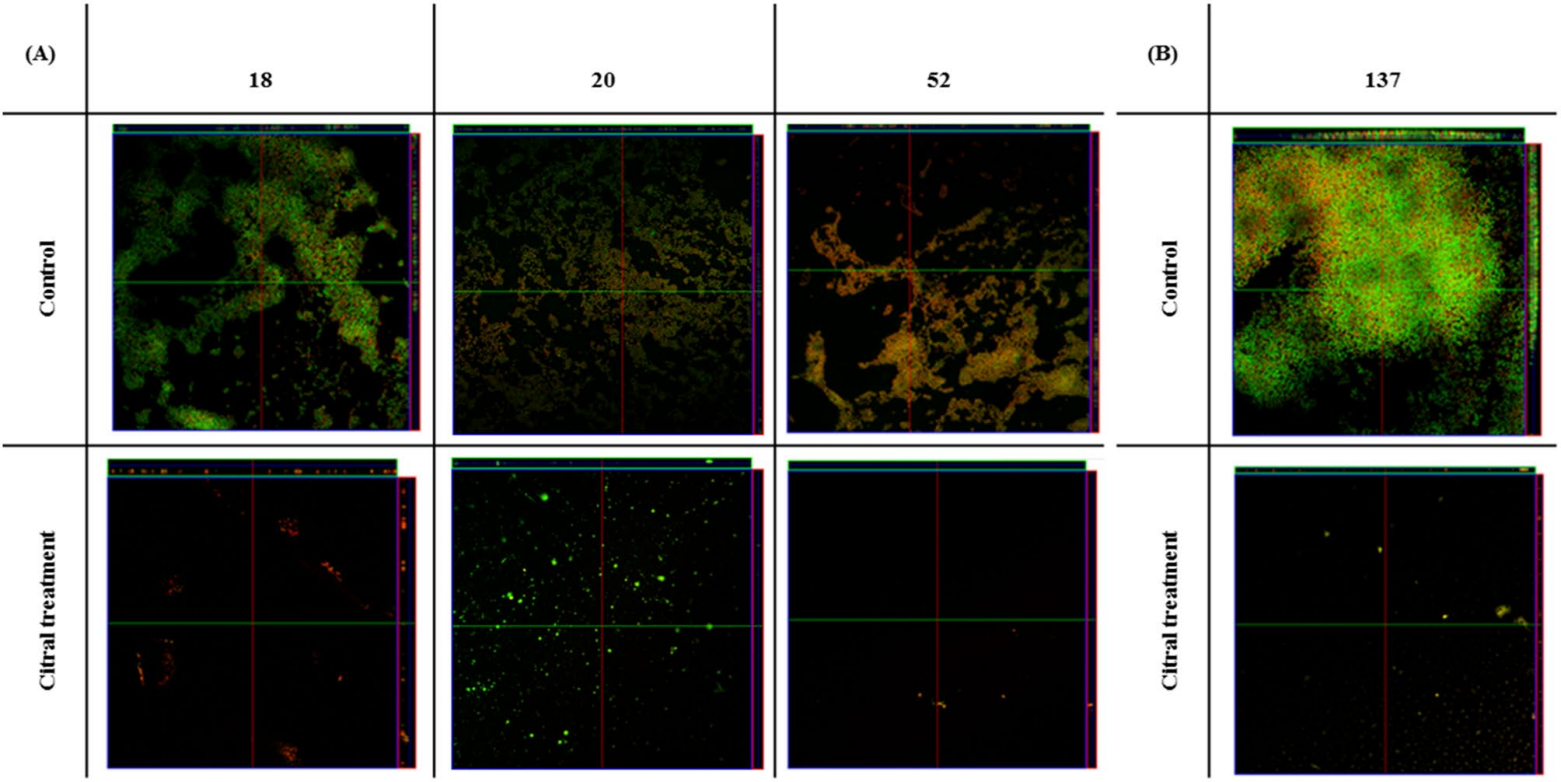
Confocal microscopy showing biofilm formation of isolated MRSA (18, 20, and 52) (control) and treated with citral 25 mg/mL (2.5%) for 24 h **(A) **and isolated MRSA (137) (control) and treated with citral 25 mg/mL (2.5%) for 48 h **(B)**. The microorganisms were marked with SYTO9 (green) and unviable with propidium iodide (red).

To confirm the results of biofilm inhibition, we visualized its structural organization using confocal microscopy. Citral treatment substantially reduced *S. aureus* biomass, with a decrease in living and dead cells (Fig. 3), consistent with what was observed in the plate biofilm.

**Figure 4:**
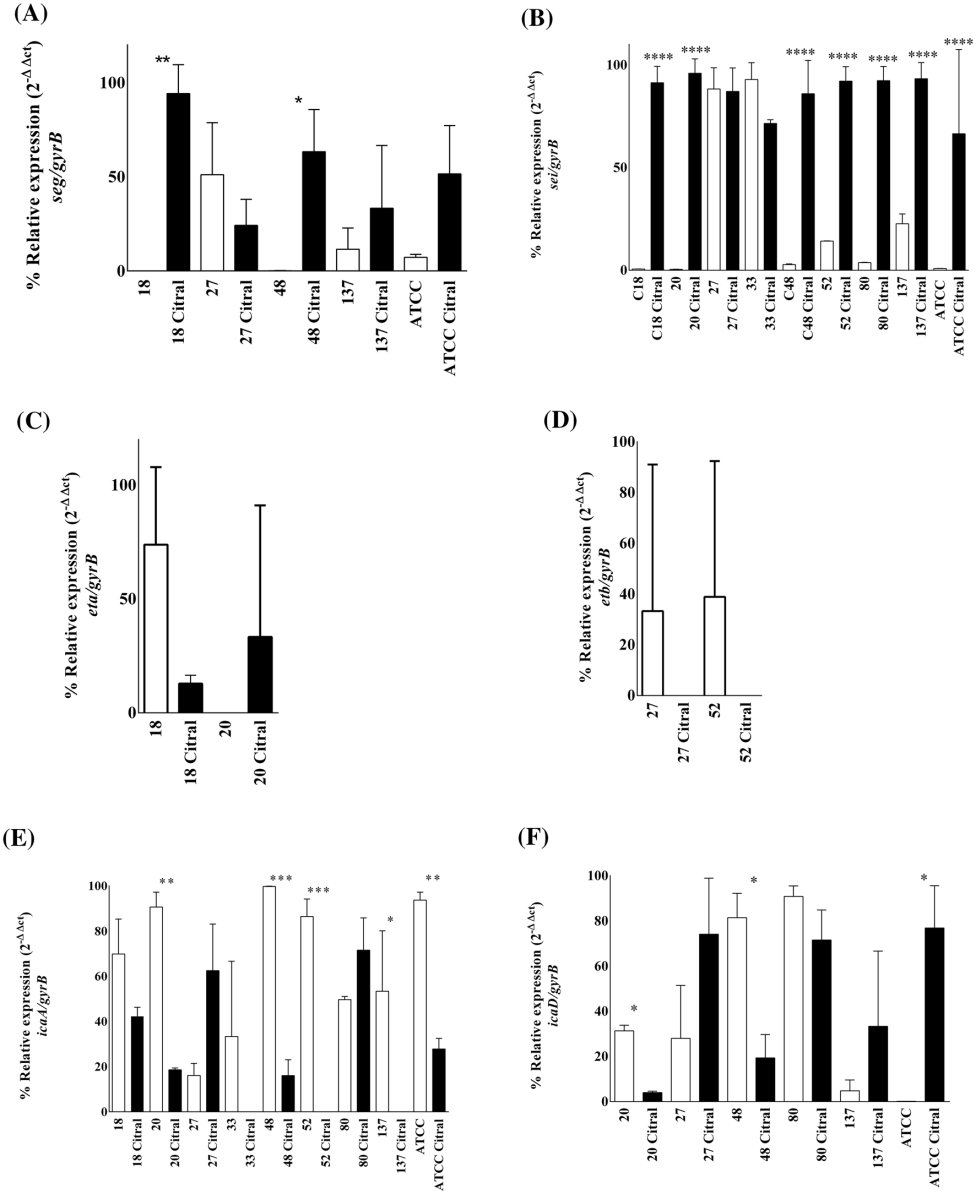
Relative expression of virulence genes *seg* **(A)**, *sei* **(B)**, *eta* **(C)**, *etb* **(D)**, *icaA* **(E)** and *icaD* **(F)** of the MRSA strains (18, 20, 27, 33, 48, 52, 80, 137) and ATCC 33591 control and with 25 mg/mL (2.5%) citral treatment. Significant difference considering *P*-value ≤ 0.05 (*), *P*-value ≤ 0.01 (**), *P*-value ≤ 0.001 (***), using Mann–Whitney one-tailed test and Kruskal–Wallis test with Dunn's test.

**Figure 5:**
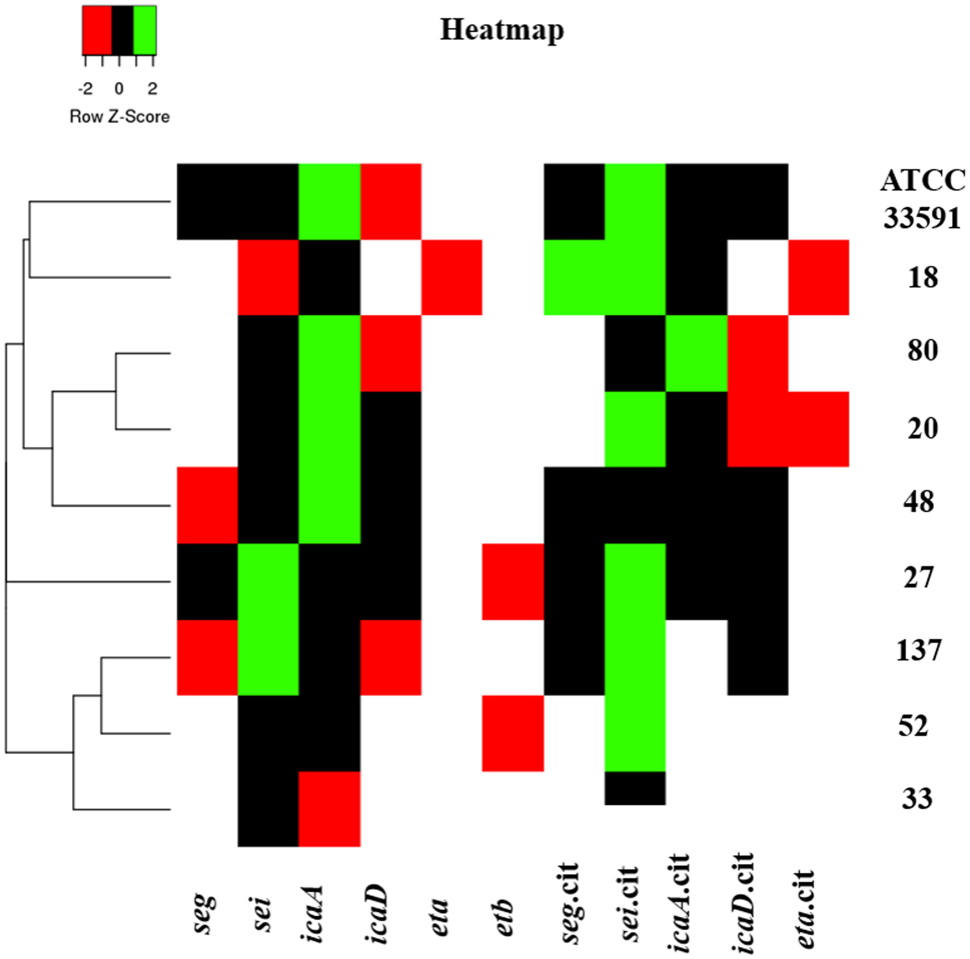
Heatmapper platform formed four clusters according of differentially expressed virulence genes *seg*, *sei*, *icaA*, *icaD, eta* and *etb* of MRSA strains (18, 20, 27, 33, 48, 52, 80, 137) and ATCC 33591 control and with 2.5% citral treatment. The green segments in the plot represent greater differential expression and the red segments represent less modulation, comparing different genes and treatment with citral.

The expression of the virulence genes of the enterotoxins *seg*, *sei*, and sej of exfoliative toxins *eta* and *etb* and of genes related to the formation of the biofilm, *icaA* and *icaD,* were evaluated, considering the characterization of the isolates regarding the virulence genes. Evaluation of the expression of the enterotoxin genes *seg* and *sei* (Fig. 4A,B), expression increased in the presence of citral, with significant results for strains 18 (100% increase) and 48 (63.7% increase) for *seg,* and 18 (93.4% increase), 20 (95.1% increase), 48 (96.8% increase) 52 (84.5% increase), 80 (95.9% increase), 137 (75.8% increase) and ATCC 33591 (98.7% increase) for *sei*. None of the MRSA strains expressed *sej*. For the expression of exfoliative toxin genes (Fig. 4C,D), *eta* and *etb*, no significant differences were found between the control group (*P* < 0.05). However, significant differences were found for bacteria and treatment with citral. Regarding the *icaA* gene (Fig. 4E), the effect of citral was observed with the underexpression of two strains, 20 (79.5% reduction), 48 (83.9% reduction), 52 (100% reduction), 137 (100% reduction) and ATCC (70.3% reduction). Isolates 20 (87.2% reduction) and 48 (76.2% reduction) treated with citral also showed a reduction in their control in the expression of the *icaD* gene (Fig. 4F). However, in the ATCC 33591 (99.8% increase) strain, it had the opposite effect with increased expression of *icaD* in the presence of the treatment. The expression of virulence was also analyzed using the heatmapper platform (http://www.heatmapper.ca/expression/), and the isolates were separated into four distinct clusters (cluster 1: ATCC 33591 and 18; cluster 2: stains 80, 20, and 48; cluster 3: strain 27; cluster 4: strains 137, 52, and 33), showing differentiated expression patterns induced by different strains of MRSA (Fig. 5).

## Discussion

*S. aureus*, MRSA, is one of the most important infectious agents because it can cause nosocomial infections, often with prolonged hospitalization, in addition to increased morbidity, mortality, and hospital costs, making bacterial resistance a major public health problem^8,22^. The use of natural products as microorganism control agents has been attracting interest in food and pharmaceutical sectors, as well as other fields, as pathogens associated with infectious diseases are developing resistance to commonly used drugs. Essential oils are secondary metabolites that contain hydrophobic compounds with the ability to easily diffuse through the cell wall of microorganisms, causing damage to the membrane, especially in fluidity and permeability, leading to the loss of intracellular substances and, consequently, their death^23,24^.

In the present study, our findings show that citral is capable of interfering with bacterial development, presenting bacteriostatic and/or bactericidal activity depending on the concentration, but the most strains and antibiotics did not show a change in antibiotic activity when citral was added. Other studies have evaluated the activity of essential oils against MRSA, including lemongrass, which has citral as one of its major components, and obtained an MIC of 0.78% and an MBC of 3.12%^25^. Another study analyzed the action of citral, linalool, decanal, and valencene on *S. aureus*, diluted with Tween 80, and observed that only citral and linalool were able to inhibit growth, with MIC values of 0.02% and 0.12%, respectively^18^. While other authors observed lower MIC (0.03%) and MBC (0.06%) values with essential oils and their main components, including citral, against *S. aureus*^26^, compared to those found in this study.

Owing to the emergence of resistance to penicillin, methicillin, and vancomycin, the options and effectiveness of antimicrobials have been reduced against *S. aureus*^27^. Thus, the use of natural products as microorganism control agents is relevant, since pathogens are becoming increasingly resistant to drugs used in clinical practices^28^. The mechanism of action of these essential oils depends on their chemical composition, and their antimicrobial activity is not the result of just one mechanism, but of various reactions that involve the entire bacterial cell^29^. It was observed that citral showed synergism, antagonism, or did not interfere with the antibiotic action, but the isolates remained resistant. Another study observed that the essential oil from the leaves of chemotype II of *Lippia alba* (MILL.) can modulate the activity of the oxacillin by synergistic or antagonistic effect in MRSA strains^30^. In addition, synergistic modulating activity was also observed for this essential oil with the antibiotics amikacin and clarithromycin, while it was indifferent with oxacillin, ciprofloxacin, and vancomycin or antagonist (clindamycin) when treating *S. aureus* ATCC 6538P^31^.

The effect of biofilm modulation is important because *S. aureus* can develop intractable infections by forming biofilms, mainly by colonizing devices such as heart valve prostheses, cardiac stimulators, contact lenses, and intravascular lines^32^. In general, the reduction in MRSA biofilm formation showed greater efficacy when the strains were treated with citral in the initial period of formation. The reduction of the fatty acid composition of the cell membrane and hydrophobicity are possible features of antibiofilm activity caused by terpenes, one of the components of this oil^33^. These components primarily target the cell wall and cytoplasmic membrane or membrane proteins, thus leading to cell death and, consequently, loss of attachment to surfaces^34^. Other authors have observed that sub-inhibitory concentrations of lemongrass essential oil and its major components, such as citral and geraniol, were able to significantly inhibit the biofilm formation of *S. aureus* strains isolated from subclinical mastitis, in addition to preformed biofilms^35^. In another study, citral inhibited biofilm formation by MRSA without affecting growth at 100 μg/mL, while greatly hampering surface adherence^36^.

As in this study, the evaluation of clove and cinnamon essential oil activity under the strain of *S. aureus* ATCC 6538, classified as strong producers of biofilm, also showed no activity on the mature biofilm^36^.

Furthremore, another study treating mature biofilms of *S. aureus* ATCC 29740, with citral at concentrations of 0.02, 0.04, and 0.08% did not report significant differences in their responses^18^. The induction of biofilm formation can occur in potentially toxic conditions for bacterial cells, such as high levels of osmolarity, oxidative stress, and in the presence of sub-inhibitory concentrations of possible treatments, among others^37^. Citral possibly has a greater effect on planktonic cells, as they are more susceptible to the action of the compound since they are not embedded in a matrix. The reduced activity of citral on the strains with mature biofilms may be related to a concentration of 25 mg/mL. Furthermore, the mass production of biofilm is directly related to the incubation time^38^.

Studies have shown the use of essential oils against susceptible and resistant methicillin strains of *S. aureus* and their action against virulence factors^38,39^. The virulence potential of different *S. aureus* isolates is determined by the presence or absence of virulence genes; each bacterial strain has a unique combination of surface proteins and regulators, and they carry a variety of mobile genetic elements coding for bacterial resistance and virulence genes, indicating frequent horizontal transfer^40^. The *icaA* and *icaD* genes, responsible for biofilm formation, were underexpressed during citral exposure in strains 20 and 48. In strain ATCC 33591, expression of *icaD* (relative to the housekeeping control gene *gyrA*), increased on exposure to citral. These results show the effect of citral on the two isolates in the deregulation of these genes, possibly contributing to reducing pathogen virulence. The icaADBC operon encodes enzymes involved in the biosynthesis of polysaccharide intercellular adhesin (PIA) or polymeric *N*-acetylglucosamine (PNAG), which play an important role in biofilm formation. It is known that the decrease in these enzymes leads to negative regulation of *icaA*, and consequently a reduction in biofilm^41^. Contrary to the results found in this study, other authors did not observe deregulation of the *icaA* and *icaD* genes in *S. aureus* with citral activity at concentrations of 0.02% and 0.04%, despite being effective for other genes^18^. Studies report the activity of essential oils through the quorum-sensing system, being a possible alternative for divergence and bacterial susceptibility^42^. It is known that in the presence of antibiotics and under certain conditions, microorganisms can present an unexpected phenotype, such as an increase in gene expression^43^. In the presence of citral, some isolates showed a significant difference compared to the positive control, increasing gene expression, such as in strains 18 and 48 for the *seg* gene, and strains 18, 20, 48, 52, 80, 137 and ATCC 33591 for the *sei* gene. These data suggest that the sub-inhibitory concentration of essential oil for some strains was not sufficient to act on the dysregulation of enterotoxin genes. Some studies have shown that at concentrations below the MIC, antibiotics can alter the gene expression of microorganisms causing infections, triggering a contrary response^44^. Another study using the essential oils of *Cinnamomum zeylanicum* and *Ocimum basilicum* observed a reduction in the expression of *sea*, *sec*, and enterotoxin genes^45^. Comparing the results obtained for biofilm structure and gene expression in MRSA 20, we noticed that after incubation with citral for 24 h, a significant reduction in biofilm formation. This finding is consistent with the results observed in the gene expression of *ica*A and *ica*D also reduced when treated with citral (Fig. 4).

## Conclusion

Our results showed that citral is also capable of modifying the biofilm produced by MRSA in the initial stage, and also exerts regulatory activity against virulence genes. It is important to emphasize the possibility of using essential oils and their major components as prophylactics or for supporting the treatment of MRSA infections, which are important for public health issues. Finally, there is a need for further studies to evaluate the activity of citral in combination with conventional antibiotics or prophylactic use, since there are few studies on the interaction between citral and MRSA.

## Materials and methods

The present study selected MRSA strains (20, 27, 33, and 52) obtained from raw human milk in the Human Milk Bank of Esaú Matos Municipal Hospital located in Vitória da Conquista, Bahia, Brazil, that were used in a previous study^46^ and MRSA strains (18, 48, 80, and 137) isolated from nasal swabs obtained from healthy children ranging from one to six years attending daycare centers located in Vitória da Conquista, Bahia, Brazil^47^. The strains used were obtained from other studies approved by the Ethics Committee of Research with Human Beings of the Multidisciplinary Health Institute campus Anísio Teixeira (CAAE no. 08730012.4.0000.5556) and 08731912.5.0000.5556 (nasal strains). All methods were performed in accordance with the relevant guidelines and regulations. MRSA ATCC 33591 was obtained from a commercial source. Each sample was plated onto plates with mannitol salt agar and incubated at 37 °C for 24 h.

### Citral

Citral was provided commercially by Sigma^®^ Aldrich. For the tests run, the citral was diluted in propylene glycol, and used in concentrations of 40 mg/mL (4%), 20 mg/mL (2%), 10 mg/mL (1%), 5 mg/mL (0.5%), 2.5 mg/mL (0.25%), 1.2 mg/mL (0.12%), and 0.6 mg/mL (0.06%), as recommended in the literature^48,49^.

### Determination of minimum inhibitory concentrations (MIC) and minimum bactericidal concentrations (MBC)

Bacterial suspension was prepared using a 0.9% sodium chloride solution, and the inoculum (1 mL) was adjusted using a spectrophotometer at 660 nm (1–5 × 10^8^ CFU/mL). MIC tests were performed by microdilution using 96-well microplates with 190 μL of the inoculum and 10 μL of citral at concentrations of 40 mg/mL (4%), 20 mg/mL (2%), 10 mg/mL (1%), 5 mg/mL (0.5%), 2.5 mg/mL (0.25%), 1.2 mg/mL (0.12%), and 0.6% (0.06%), which were used as negative controls for brain heart infusion. The plates were incubated at 37 °C for 24 h. The MIC was considered the lowest concentration of citral, which showed no visible bacterial growth. To determine the MBC, suspensions used in the MIC test were inoculated onto BHI agar plates. All experiments were performed in triplicate with three independent repetitions^50^.

### Determination of subinhibitory dose

To verify the modulation of virulence factors without influencing the viability of microorganisms, after the definition of MIC and MBC, the effect of a subinhibitory dose on bacterial growth was evaluated. The growth curves of MRSA alone and MRSA treated with citral at concentrations of 40 mg/mL (4%), 20 mg/mL (2%), 10 mg/mL (1%), 5 mg/mL (0.5%), 2.5 mg/mL (0.25%), 1.2 mg/mL (0.12%), and 0.6 mg/mL (0.06%) was performed in a closed system, with an inoculum of 1–5 × 10^8^ CFU/mL, at 37 °C. The microbial growth curve was determined every hour for 24 h by spectrophotometry with absorbance at 660 nm (Bel Photonics Spectrophotometer UV–VIS M51), and the viable cell count was determined by serial dilutions and seeding on BHI every hour^50^.

### Evaluation of modulating activity: citral interference in antibiotics

To evaluate citral as a modulator of antibiotic action, the evaluation was performed with six antibiotics, including ampicillin (10 µg), ciprofloxacin (5 μg), clindamycin (10 μg), erythromycin (15 μg), oxacillin (1 μg), and tetracycline (30 μg) associated with citral (5 mg/mL, 0.5%), and the lowest MIC found in the tested strains. Antibiotic susceptibility testing was performed using the disc diffusion method according to the CLSI^51^. The MIC value was determined according to CLSI^51^. The experiments were performed in triplicate with three independent repetitions.

### Inhibition of biofilm formation

The ability of MRSA to form biofilms following treatment with 25 mg/mL (2.5%) citral was analyzed according to previously proposed methods^52^. Biofilm assays were performed in 96-well polystyrene microplates, using trypticase soy broth (TSB/Difco) with 1% (w/v) glucose (TSB-1% Glc). Cultures of MRSA strains (5 mL) were incubated in a shaker at 250 rpm at 37 °C for 18 h, after which they were diluted 1:100 in TSB-1% Glc and 200 μL were inoculated into each well and incubated at 37 °C for 24 h. Supernatants were removed from each well and biofilms were washed twice with PBS, dried, fixed at 65 °C for 1 h, stained with crystal violet 1%, and gently washed twice with PBS. MRSA strains were tested using citral at a concentration 10 times greater than that obtained in the subinhibitory dose^53^. Two phases of biofilm formation were evaluated: early phase 0 to 24 h after inoculation (the period of microcolony formation) and mature phase 24 h after inoculation (the period after thin biofilm formation)^54^. The experiment was carried out in quadruplicate with two independent experiments. Biofilm production was compared to that of *Streptococcus pyogenes* ATCC 75194 (A_492nm_ = 0.07). The biofilm formation index (BFI) was calculated as follows: BFI = x/y (where "x” is the optical density at A492 nm of the biofilm and *y* is the optical density at 492 nm of *Streptococcus pyogenes* [0.07]). Furthermore, to confirm the differences between biofilm phenotypes, confocal laser scanning microscopy (CLSM) was used to obtain structural images. Here, the biofilm assays were performed in the same way, but after being fixed, the bacterial cells were stained with 25 nM SYTO9 and propidium iodide (Live/Dead Bacteria, Invitrogen, Brazil) for 15 min in the dark. The stain was gently removed, and biofilms were observed using a CLSM (Carl Zeiss LSM 510, Germany) equipped with an argon laser (488 nm, and 2 helium/neon 543 nm wavelengths) to visualize the luminescence of fluorochromes^55^.

### Virulence factor expression modulation

To determine a possible modulating effect of citral on different virulence genes, a sub-inhibitory concentration of 2.5 mg/mL (0.25%) was used and incubated in a shaker at 37 °C for 4 h (when all strains reached their exponential growth). Total mRNA was extracted using the PureLink™ RNA Mini kit (Life Technologies) and quantified by spectrophotometry (NanoDrop 2000/2000c Spectrophotometer, Thermo Fisher Scientific, Waltham, MA, US). cDNA was obtained using the SuperScript III Reverse Transcriptase Kit (Invitrogen). To detect genes related to virulence *sec*, *sei*, *sej*^56^, *icaD*, *icaA*^57^, *eta* and *etb*^58^, qPCR was used, using primers and conditions previously described. The gyrB gene^59^ was used as an endogenous control. This protocol included, in a final reaction volume of 27 μL, which included 12.5 μL of SYBR® Green Master Mix (Applied Biosystems), 1 μL (13 μM) of each primer, 10.5 μL of H_2_O RNAse free and 2 μL of cDNA. Gene expression data were analyzed using the 2^-ΔΔCT^ method and analyzed in triplicate.

### Statistical analysis

GraphPad Prism 6.0 (GraphPad Software, San Diego, CA, USA) was used for analysis. The following nonparametric tests were used: Kruskal–Wallis with Dunn's test (when evaluating more than two groups) and a Mann–Whitney one-tailed test (when evaluating two groups). Statistical differences were considered significant at *P* < 0.05, using a 95% confidence interval. Citral interference in antibiotics and modulation of virulence factor expression were analyzed using the heatmapper platform (http://www.heatmapper.ca/expression/) and represented as a heatmap. Unsupervised hierarchical grouping was performed using the average distance and Euclidean distance as metrics.

### Ethics declarations and approval for human experiments

Ethics approval and consent were deemed unnecessary in this study, according to the Animal Ethics Committee (AEC) of the Multidisciplinary Health Institute, Federal University of Bahia. The strains used were obtained from other studies after approval by the Ethics Committee of Research with Human Beings of the Multidisciplinary Health Institute campus Anísio Teixeira (CAAE no. 08730012.4.0000.5556) and 08731912.5.0000.5556 (nasal strains). For nasal samples, informed consent was obtained from the parents or guardians.

## Data Availability

The datasets used and/or analyzed during the current study are available from the corresponding author upon reasonable request.

## References

[1] Cassettari VC, Strabelli T, Medeiros EAS (2005). *Staphylococcus aureus* bacteremia: What is the impact of oxacillin resistance on mortality?. Braz. J. Infect. Dis..

[2] Gelatti LC (2009). Sepsis due to community-acquired methicillin-resistant *Staphylococcus aureus* in southern Brazil. Rev. Soc. Bras. Med. Trop..

[3] Brown AF, Leech JM, Rogers TR, Mcloughlin RM (2014). *Staphylococcus aureus* colonization: Modulation of host immune response and impact on human vaccine design, Irlanda. Front. Immunol..

[4] Ünal N, Askar Ş, Macun HC, Sakarya F, Altun B, Yıldırım M (2012). Panton-Valentine leukocidin and some exotoxins of *Staphylococcus aureus* and antimicrobial susceptibility profiles of staphylococci isolated from milks of small ruminants. Trop. Anim. Health Prod..

[5] Stefani S (2012). Meticillin-resistant *Staphylococcus aureus* (MRSA): Global epidemiology and harmonisation of typing methods. Int. J. Antimicrob. Agents.

[6] Gordon RJ, Lowy FD (2008). Pathogenesis of methicillin-resistant *Staphylococccus aureus* infection. Clin. Infect. Dis..

[7] Stryjewski ME, Corey GR (2014). Methicillin-resistant *Staphylococcus aureus*: An evolving pathogen. Clin. Infect. Dis..

[8] Turner NA (2019). Methicillin-resistant *Staphylococcus aureus*: An overview of basic and clinical research. Nat. Rev. Microbiol..

[9] Wang Y (2018). Effect of cinnamaldehyde and citral combination on transcriptional profile, growth, oxidative damage and patulin biosynthesis of *Penicillium expansum*. Front. Microbiol..

[10] Spanu V (2012). Virulence factors and genetic variability of *Staphylococcus aureus* strains isolated from raw sheep’s cheese. Int. J. Food Microbiol..

[11] Mendonça ECL (2012). Caracterização fenogenotípica da resistência antimicrobiana em *Staphylococcus* spp. isolados de mastite bovina. Pesq. Vet. Bras..

[12] Singh G, Maurya S, deLampasona MP, Catalan CAN (2007). A comparison of chemical, antioxidant and antimicrobial studies of cinnamon leaf and bark volatile oils, oleoresins and their constituents. Food Chem. Toxicol..

[13] Bakkali F, Averbeck S, Averbeck D, Idaomar M (2008). Biological effects of essential oils-a review. Food Chem. Toxicol..

[14] Calo JR, Crandall PG, O’Bryan CA, Ricke SC (2015). Essential oils as antimicrobials in food systems - A review. Food Control.

[15] Dudai N, Weinstein Y, Krup M, Rabinski T, Ofir R (2005). Citral is a new inducer of caspase-3 in tumor cell lines. Planta Med..

[16] Saddiq AA, Khayyat SA (2010). Chemical and antimicrobial studies of monoterpene: Citral. Pestic. Biochem. Physiol..

[17] Prins CL, Freitas SP, Campostrini E, Gravina GA, Reis FO (2008). Efeitos de confinamento do sistema radicular sobre capim-limão (*Cymbopogon citratus*). Rev. Cien. Agronom..

[18] Federman C, Ma C, Biswas D (2016). Major components of orange oil inhibit *Staphylococcus aureus* growth and biofilm formation, and alter its virulence factors. J. Med. Microbiol..

[19] Irkin R, Korukluoglu M (2009). Effectiveness of *Cymbopogon citratus* L. essential oil to inhibit the growth of some filamentous fungi and yeasts. J. Med. Food.

[20] Tyagi AK, Malik A (2010). Liquid and vapour-phase antifungal activities of selected essential oils against *Candida albicans*: Microscopic observations and chemical characterization of *Cymbopogon citratus*. BMC Complement. Altern. Med..

[21] Martins HB (2017). Anti-inflammatory activity of the essential oil citral in experimental infection with *Staphylococcus aureus* in a model air pouch. Evid. Based Complement. Alternat. Med..

[22] da Silva JG, Souza IA, Higino JS, Siqueira-Junior JP, Pereira JV, Pereira MdSV (2007). Atividade antimicrobiana do extrato de *Anacardium occidentale* Linn. em amostras multiresistentes de *Staphylococcus aureus*. Rev. Bras. Farmacogn..

[23] Valeriano C, Piccoli RH, Cardoso MG, Alves E (2012). Atividade antimicrobiana de óleos essenciais em bactérias patogênicas de origem alimentar. Rev. Bras. Plant. Med..

[24] Pu S, Wang F, Ge B (2011). Characterization of toxin genes and antimicrobial susceptibility of *Staphylococcus aureus* isolates from Louisiana retail meats. Foodborne Pathog. Dis..

[25] Sroiphetcharat S, Sukplang P, Thongmee A (2017). *In vitro* investigation of antimicrobial activity of essential oil against methicillin resistant *Staphylococcus aureus* (MRSA). BHST.

[26] Santos CHS, Piccoli RH, Tebaldi VMR (2017). Antimicrobial activity of the essential oils and isolated compounds on the hospital-borne and foodborne pathogens. Rev Inst Adolfo Lutz. São Paulo.

[27] Feng Y (2008). Evolution and pathogenesis of *Staphylococcus aureus*: lessons learned from genotyping and comparative genomics. FEMS Microbiol. Rev..

[28] Mesa-Arango AC (2009). Citral and carvone chemotypes from the essential oils PF Colombian Lippia Alba (Mill.) N.E. Brown: Composition, cytotoxicity and antifungal activity. Mem. Inst. Oswaldo Cruz.

[29] Nazzaro F, Fratianni F, De Martino L, Coppola R, De Feo V (2013). Effect of essential oils on pathogenic bacteria. Pharmaceuticals (Basel).

[30] Rocha, L. Q. Interferência do óleo essencial de folhas do quimiotipo II de *Lippia alba* (MILL.) NE BROWN na atividade antimicrobiana da oxacilina sobre *Staphylacoccus aureus* oxacilina-resistente. in 127 f. *Dissertação (Mestrado em Ciências Farmacêuticas)—Universidade Federal do Ceará*. (Faculdade de Farmácia, Odontologia e Enfermagem, Fortaleza, 2012).

[31] Sales GWP, Batista AHM, Rocha LQ, Nogueira NAP (2014). Efeito antimicrobiano e modulador do óleo essencial extraído da casca de frutos da *Hymenaea courbaril L*. Rev. Ciênc. Farm. Básica Apl..

[32] McCarthy H, Rudkin JK, Black NS, Gallagher L, O’Neill E, O’Gara JP (2015). Methicillin resistance and the biofilm phenotype in *Staphylococcus aureus*. Front. Cell. Infect. Microbiol..

[33] de Carvalho CC, da Fonseca MM (2007). Preventing biofilm formation: Promoting cell separation with terpenes. FEMS Microbiol. Ecol..

[34] Silva LN, Zimmer KR, Macedo AJ, Trentin DS (2016). Plant natural products targeting bacterial virulence factors. Chem. Rev..

[35] Aiemsaard J, Aiumlamai S, Aromdee C, Taweechaisupapong S, Khunkitti W (2011). The effect of lemongrass oil and its major components on clinical isolate mastitis pathogens and their mechanisms of action on *Staphylococcus aureus* DMST 4745. Res. Vet. Sci..

[36] Valliammai A (2020). Proteomic profiling unveils citral modulating expression of IsaA, CodY and SaeS to inhibit biofilm and virulence in methicillin-resistant *Staphylococcus aureus*. Int. J. Biol. Macromol..

[37] Nuryastuti T (2009). Effect of cinnamon oil on icaA expression and biofilm formation by *Staphylococcus epidermidis*. Appl. Environ. Microbiol..

[38] Espina L, Pagán R, López D, García-Gonzalo D (2015). Individual constituents from essential oils inhibit biofilm mass production by multi-drug resistant *Staphylococcus aureus*. Molecules.

[39] Jafri H, Husain FM, Ahmad I (2014). Therapeutic sciences antibacterial and antibiofilm activity of some essential oils and compounds against clinical strains of *Staphylococcus aureus*. J. Biomed. Ther. Sci..

[40] Lloyd JMDH, Lindsay JA (2008). *Staphylococcus aureus* host specificity: Comparative genomics of human versus animal isolates by multistrain microarray L-sung. J. Microbiol..

[41] Ma Y (2012). Novel inhibitors of *Staphylococcus aureus* virulence gene expression and biofilm formation. PLoS ONE.

[42] Rutherford ST, Bassler BL (2012). Bacterial quorum sensing: Its role in virulence and possibilities for its control. Cold Spring Harb. Perspect. Med..

[43] Joo HS, Chan JL, Cheung GYC, Otto M (2010). Subinhibitory concentrations of protein synthesis-inhibiting antibiotics promote increased expression of the agr virulence regulator and production of phenol-soluble modulin cytolysins in community-associated methicillin-resistant *Staphylococcus aureus*. Antimicrob. Agents Chemother..

[44] Davies J, Spiegelman GB, Yim G (2006). The world of subinhibitory antibiotic concentrations. Curr. Opin. Microbiol..

[45] Azizkhani M, Parsaeimehr M (2015). Effects of *Cinnamomum zeylanicum* and *Ocimum basilicum* essential oils on the growth of *Staphylococcus aureus* ATCC 29213 and gene expression of enterotoxins A, C and E. J. Essent. Oil Res..

[46] de Almeida JB (2014). Detection, antibiotic resistance, and pathogenicity of staphylococci in samples from a Brazilian human milk bank. Breastfeed. Med..

[47] de Carvalho SP (2017). Community-acquired methicillin-resistant *Staphylococcus aureus* carrying SCCmec type IV and V isolated from healthy children attending public daycares in northeastern Brazil. Braz. J. Infect. Dis..

[48] Adukwu EC, Allen SC, Phillips CA (2012). The anti-biofilm activity of lemongrass (*Cymbopogon flexuosus*) and grapefruit (*Citrus paradisi*) essential oils against five strains of *Staphylococcus aureus*. J. Appl. Microbiol..

[49] Adukwu EC, Bowles M, Edwards-Jones V, Bone H (2016). Antimicrobial activity, cytotoxicity and chemical analysis of lemongrass essential oil (*Cymbopogon flexuosus*) and pure citral. Appl. Microbiol. Biotechnol..

[50] Yatsuda R (2005). Effects of *Mikania* genus plants on growth and cell adherence of mutans streptococci. J. Ethnopharmacol..

[51] Clinical and Laboratory Standards Institute. *Performance Standards for Antimicrobial Susceptibility Testing; Twenty-Second Informational Supplement. CLSI Document m100- S22*. (Clinical and Laboratory Standards Institute, 2020).

[52] Oliveira PS (2014). Isolation, pathogenicity and disinfection of *Staphylococcus aureus* carried by insects in two public hospitals of Vitória da Conquista, Bahia. Brasil. Braz. J. Infect. Dis..

[53] Lewis K (2001). Riddle of biofilm resistance. Antimicrob. Agents Chemother..

[54] Takahashi N, Ishihara K, Kato T, Okuda K (2007). Susceptibility of Actinobacillus actinomycetemcomitans to six antibiotics decreases as biofilm matures. J. Antimicrob. Chemother..

[55] Coelho LR (2008). agr RNAIII divergently regulates glucose-induced biofilm formation in clinical isolates of *Staphylococcus aureus*. Microbiology (Reading).

[56] Lee YD, Moon BY, Park JH, Chang HI, Kim WJ (2007). Expression of enterotoxin genes in *Staphylococcus aureus* isolates based on mRNA analysis. J. Microbiol. Biotechnol..

[57] Korem M, Gov Y, Rosenberg M (2010). Global gene expression in *Staphylococcus aureus* following exposure to alcohol. Microb. Pathog..

[58] Johnson WM (1991). The detection of enterotoxins and toxic shock syndrome toxin genes in *Staphylococcus aureus* by polymerase chain reaction. J. Clin. Microbiol..

[59] Abdelhady W (2015). Early agr activation correlates with vancomycin treatment failure in multi-clonotype MRSA endovascular infections. J. Antimicrob. Chemother..

